# *Neisseria gonorrhoeae* Meningitis in Pregnant Adolescent

**DOI:** 10.3201/eid1410.080118

**Published:** 2008-10

**Authors:** Maria C. Martín, Francisco Pérez, Alfonso Moreno, Alberto Moral, Miguel A. Alvarez, Francisco J. Méndez, Fernando Vázquez

**Affiliations:** Instituto de Productos Lácteos de Asturias, Spain (M.C. Martín, M.A. Alvarez); Hospital Universitario Central de Asturias, Oviedo, Spain (F. Pérez, A. Moreno, A. Moral, F.J. Méndez); Facultad de Medicina Area de Microbiología, Oviedo (F.J. Méndez, F. Vázquez); Hospital Monte Naranco, Oviedo (F. Vázquez)

**Keywords:** Neisseria gonorrhoeae, meningitis, letter

**To the Editor:** Dissemination is a rare complication of gonococcal infection and has been observed in 0.5%–3% of patients (*1*). We describe a new case in a pregnant adolescent infected with a strain resistant to ciprofloxacin and tetracycline.

A 14-year-old girl of Ecuadorian descent, 24 weeks pregnant, sought treatment after a 48-hour history of holocrania cephalea, fever, nausea, and vomiting. She had returned a few days before from a holiday trip to Quito, Ecuador, and had not taken any antimicrobial drugs. She was admitted to hospital with a temperature of 40ºC and neck stiffness. Results of a neurologic examination were otherwise normal.

Laboratory blood tests at hospital admission showed a leukocyte count of 13,400 cells/mm^3^ (with a mature neutrophil count of 87%), hemoglobin of 10.8 g/dL, and a platelet count of 611,000 cells/mm^3^; electrolyte levels and results of liver function tests were normal, but a total cholesterol count of 267 mg/dL and a triglyceride level of 440 mg/dL were found. The level of C-reactive protein was elevated (17.90 mg/dL). Coagulation factors were within normal values, with the exception of a fibrinogen value of 917 mg/dL. Levels of complement components C2, C3, C6, C7, C8, and H factor were greatly elevated. The cerebrospinal fluid (CSF) sample obtained in the casualty ward had a leukocyte count of 5,000 cells/mm^3^ (90% neutrophils, 10% lymphocytes), with glucose and protein levels of 20 mg/dL and 207 mg/dL, respectively. The patient received a 2-week course of intravenous cefotaxime (2 g every 4 h) and recovered without sequelae.

Gram smear of the CSF sediment was consistent with purulent meningitis. Culture on chocolate agar showed scarce growth of small, colorless mucoid colonies that showed positive reactions in oxidase and catalase tests. Gram stain of colony smears showed gram-negative diplococci and the API NH system (bioMérieux, Marcy l’Etoile, France) classified the bacterium as *Neisseria* *gonorrhoeae* (with a score of 87%)*.* The result of the Phadebact Monoclonal GC test (Boule Diagnostics, Boule, Sweden) was positive. Cultures of endocervix, rectal, and pharyngeal samples obtained before treatment were negative. The strain was β-lactamase negative by chromogenic cephalosporin method (BBL Cefinase, Cockeysville, MD, USA) and classified as serogroup IA *rst* by agglutination with specific antisera (Neisseria Reference Center, Majadahonda, Madrid, Spain). The strain auxogroup, determined in modified Heckels media, (*2*) was arginine-hypoxanthine-uracil (AHU). Plasmid profile was obtained by standard alkaline DNA extraction (*3*) and showed a plasmid of ≈4.7 Md. The isolate was susceptible to penicillin (0.12 µg/mL) and cefotaxime (0.050 µg/mL) and resistant to tetracycline (16 µg/mL) and ciprofloxacin (4 µg/mL) by the agar dilution method (*4*).

Final identification of the bacterium was achieved by sequencing the 16S rRNA gene. The primers pA 5′-AGAGTTTGATCCTGGCTCAG-3′ and pH* 5′-AAGGAGGTGATCCAGCCGCA-3′ (Sigma-Genosys, Haverhill, England), as described by Edwards et al. (*5*), were used for amplification of 16S rRNA sequences. The PCR product was directly sequenced, and the nucleotide sequence matched the 16S rRNA gene of *N. gonorrhoeae* deposited in databases. The partial sequence of the 16S rRNA gene of *N. gonorrhoeae* determined in this study has been deposited in the European Molecular Biology Laboratory database under accession no. AM921674.

Neurologic manifestations of gonorrhea were observed as early as 1805 (*6*). However, the first well-documented case of gonococcal meningitis was not reported until 1922 (*7*) and so far, only ≈24 cases have been reported since 1922. In the preantimicrobial drug era, disemminated gonococcal infection (DGI) predominantly affected men (78%) but now is seen most frequently in women (97%) (*1*).

Other studies have listed factors that may facilitate spread of asymptomatic gonococcal infection, such as pregnancy (*1**,**8*), menstruation, viral hepatitis, differences in virulence between strains of gonococci, and host immunologic differences. The role of immunosuppressive conditions such as alcoholism or pregnancy remains unclear (*9*).

Knapp and Holmes (*10*) reported that 89% of *N. gonorrhoeae* isolated from patients with DGI were AHU auxotypes; proline dependence was also associated with DGI. These isolates were prevalent in Scandinavia and areas of the United States (Pacific Northwest, Minnesota, Wisconsin) with large Scandinavian-heritage populations. Isolated frequently in the 1970s, these strains are rarely isolated now. In other geographic areas, particularly South America, isolation of this type of strain is unusual.

In the past, all isolates recovered from patients with meningitis were susceptible to penicillin and tetracycline. Strains highly susceptible to penicillin generally require AHU for growth (*10*). Nevertheless, in the antimicrobial drug era, an increase in strains with an intermediate susceptibility to penicillin and a less invasive nature has been detected. More recently, penicillin-resistant strains recovered from patients with DGI and arthritis have emerged.

Gonococcal meningitis is a milder disease than meningitis caused by the pneumococcus or meningococcus because 6 of 9 patients seen before 1938 who were not treated with antimicrobial drugs survived (*10*) with no reported relapses or major neurologic sequelae, as in this case. In summary, we report an infrequent case of gonococcal meningitis in a pregnant adolescent with good clinical evolution and without sequelae or complications in her pregnancy.
